# Simultaneous Adsorption of Malachite Green and Methylene Blue Dyes in a Fixed-Bed Column Using Poly(Acrylonitrile-Co-Acrylic Acid) Modified with Thiourea

**DOI:** 10.3390/molecules25112650

**Published:** 2020-06-07

**Authors:** Abel Adekanmi Adeyi, Siti Nurul Ain Md Jamil, Luqman Chuah Abdullah, Thomas Shean Yaw Choong, Kia Li Lau, Nor Halaliza Alias

**Affiliations:** 1 Department of Chemical and Environmental Engineering, Faculty of Engineering, Universiti Putra Malaysia, UPM Serdang 43400, Malaysia; abeladeyi@abuad.edu.ng (A.A.A.); chuah@upm.edu.my (L.C.A.); csthomas@upm.edu.my (T.S.Y.C.); laukiali@hotmail.com (K.L.L.); norhalaliza@gmail.com (N.H.A.); 2 Department of Chemical and Petroleum Engineering, College of Engineering, Afe Babalola University Ado-Ekiti, ABUAD, KM. 8.5, Afe Babalola Way, Ado-Ekiti PMB 5454, Nigeria; 3 Department of Chemistry, Faculty of Science, Universiti Putra Malaysia, UPM Serdang 43400, Malaysia; 4 Centre of Foundation Studies for Agricultural Science, Universiti Putra Malaysia, UPM Serdang 43400, Malaysia; 5 Institute of Tropical Forestry and Forest Products (INTROP), Universiti Putra Malaysia, UPM Serdang 43400, Malaysia; 6 Faculty of Chemical Engineering, Universiti Teknologi MARA, Shah Alam 40450, Selangor, Malaysia

**Keywords:** binary system, fixed-bed, thiourea-modified poly(acrylonitrile-co-acrylic acid), malachite green, methylene blue

## Abstract

Proper remediation of aquatic environments contaminated by toxic organic dyes has become a research focus globally for environmental and chemical engineers. This study evaluates the adsorption potential of a polymer-based adsorbent, thiourea-modified poly(acrylonitrile-*co*-acrylic acid) (T-PAA) adsorbent, for the simultaneous uptake of malachite green (MG) and methylene blue (MB) dye ions from binary system in a continuous flow adsorption column. The influence of inlet dye concentrations, pH, flow rate, and adsorbent bed depth on adsorption process were investigated, and the breakthrough curves obtained experimentally. Results revealed that the sorption capacity of the T-PAA for MG and MB increase at high pH, concentration and bed-depth. Thomas, Bohart-Adams, and Yoon-Nelson models constants were calculated to describe MG and MB adsorption. It was found that the three dynamic models perfectly simulate the adsorption rate and behavior of cationic dyes entrapment. Finally, T-PAA adsorbent demonstrated good cyclic stability. It can be regenerated seven times (or cycles) with no significant loss in adsorption potential. Overall, the excellent sorption capacity and multiple usage make T-PAA polymer an attractive adsorbent materials for treatment of multicomponent dye bearing effluent in a fixed-bed column system.

## 1. Introduction

The treatment of dye-bearing wastewater has become subject of great concern nowadays, due to environmental pollution and its adverse effect on public health. Cationic dyes are highly soluble in water and produce bright shining color. They are released as effluent water from industries such as paint, textile, printing, pharmaceutical, rubber, leather, food, and cosmetics [1]. Cationic dyes and their breakdown products are mutagenic, carcinogenic, and even toxic at trace level [2,3,4]. The separation of these organic dyes from industrial effluents is therefore germane, not only in terms of water resource protection, but also for the preservation of ecological environment and human health.

The most widely applied technique for the removal of dyes and other emerging contaminants from water is adsorption process, due to simple operating procedure, effectiveness and regeneration capability. Besides, adsorption is use for wide range of pollutants even at low pollutant concentrations in batch and continuous process mode [2,5]. Fixed-bed adsorption column are extensively used for water purification and pollution control. Significant volumes of contaminated water are treated in a short time of column operation. Moreover, fixed-bed columns are vital in the determination of key adsorption test parameters such as breakthrough and saturated times, which are used to assess process efficiency and it industrial applicability [6].

Although, the effectiveness of adsorption process is largely dependent on the adsorbent materials and separation unit designation [7]. A good adsorbent material should be chemically stable with outstanding mechanical property under severe conditions. This enhanced its regeneration for multiple usage. Second, the adsorbent should possess accessible pores with large contact area to facilitate mass diffusion within porous media. Its porous surface must contain functional groups which promotes or determines selectivity and sorption capacity [8,9]. Numerous materials have been assessed for the uptake of cationic dyes from aquatic environments.

Adsorption studies are often limited to batch experiments with single component contaminant [10,11,12], which do not provide adequate scale-up data for possible multicomponent industrial scale wastewater treatment. A knowledge gap exists in adsorption behavior in dynamic systems, affirming the necessity of this work. This present paper presents a continuation of our previous works, which prepared and identified thiourea-modified poly(acrylonitrile-*co*-acrylic acid) as a potential adsorbent, for single and binary batch adsorption of malachite green and methylene blue [13,14,15,16].

Few works have reported the binary dye adsorption using continuous flow conditions, which are more relevant in large scale textile wastewater treatment. Thus, the main focus of this study was to evaluate the binary adsorption of malachite green (MG) and methylene blue (MB) from aqueous solution in a fixed-bed column system. The influence of column operation variables (pH, initial dye concentration, bed depth, and flow rate) on binary dye adsorption were presented. The dynamic of the adsorption process were also modeled with the Thomas, Bohart-Adams, and Yoon-Nelson models to predict the column performance.

## 2. Materials and Methods

### 2.1. Chemicals

Acrylonitrile (AN), acrylic acid (AA) (Acros Organics, New Jersey, USA), aluminum oxide (MERCK, Darmstadt, Germany), potassium persulphate, sodium bisulfate, thiourea (TU) (R&M Chemicals, Essex, UK), hydrochloric acid, sodium hydroxide (R&M Chemicals, Essex, UK), methanol, and ethanol were purchased from Systerm ChemAR (Shah Alam, Malaysia). All chemical reagents were analytical grade, used without further purification except AN and AA purified by passing it through Al_2_O_3_ in a glass column. Fine acid washed sea sand was purchased from Fisher Chemicals (Thermo Fisher Scientific, Waltham Massachusetts, UK).

### 2.2. Synthesis of Thiourea Modified Poly(Acrylonitrile-Co-Acrylic Acid) (T-PAA)

Free radical polymerization (redox polymerization) of AN and AA was performed at 60 °C under N_2_ gas in a three-necked round-bottomed flask, fitted with a water condenser. The monomers feed ratio AN:AA was 97:3 (vol/vol). The reaction medium, 200 mL deionized (DI) water was purged firstly with N_2_ gas for 30 min at 40 °C. Then, 0.275 mol of AN and 0.029 mol of AA were added into the reaction medium followed by 2.16 g of potassium persulphate (KPS) and 2.09 g of sodium bisulfate (SBS) as initiators. The solution was stirred mechanically at agitation speed of 200 rpm by using egg-shaped magnetic stirrer, and purged with N_2_ gas to promote redox polymerization. The polymerization reaction was allowed for 2 h. The polymer formed was precipitated in methanol for 1 h. The polymer was filtered and washed successively with methanol and DI water. The polymer, poly(AN-*co*-AA) was dried in vacuo at 45 °C until a constant weight was obtained [17,18].

**Figure 1:**
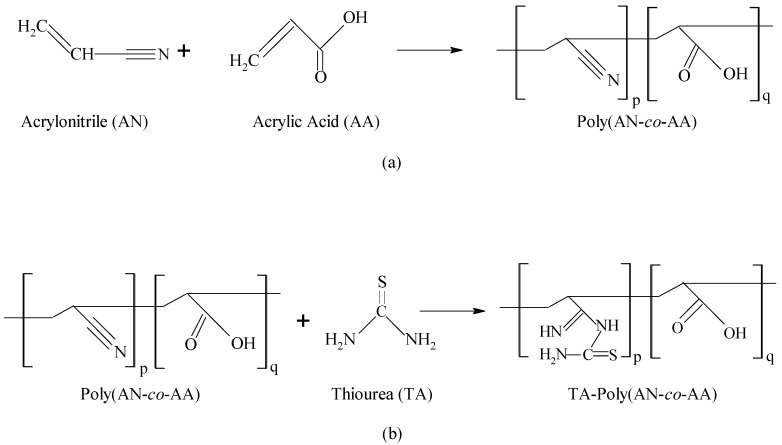
Synthesis of poly(acrylonitrile-*co*-acrylic acid) (poly(AN-*co*-AA) (**a**) and its modification with thiourea (**b**) [13].

For surface modification, 6.0 g of thiourea and ethanol/deionized water (1:2 *v/v*) were mixed and stirred at 200 rpm for 30 min at 70 °C temperature. Then, 5.0 g of poly(AN-*co*-AA) was added to the solution, and stirred at 200 rpm for 5 h at 100 °C. Then, the resulting solids thiourea-modified poly(AN-*co*-AA) (T-PAA), rinsed in ethanol/DI water solution, filtered, and dried at 50 °C to constant weight. The synthesis and modification route are represent by Figure 1.

### 2.3. Preparation of Binary Dye Solution and Determination of Dye Concentration

**Figure 2:**
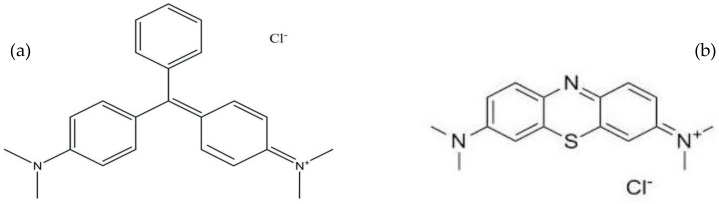
Molecular structure of (**a**) malachite green and (**b**) methylene blue.

The performance of T-PAA adsorbent towards binary cationic dye solution was evaluate using malachite green (MG) and methylene blue (MB). The two dyes were purchased from Acros Organics, New Jersey and used without purification. Their molecular structures and general properties are shown in Figure 2 and Table 1, respectively.

The single component stock solution (1000 mg/L) of MG and MB were prepared by dissolving 1.0 g of each dye in 1000 mL of double distilled water in a volumetric flask, respectively. Prior to each adsorption study, solutions of various dye concentrations (as presented in Table 2) were prepared via fresh dilution of the stock solution with distilled water. The mixing ratio of each binary solution sample was 1:1; 100 mL of every cationic dye solution contains a mixture of 50 mL of MG and 50 mL of MB.

Calibration of cross-interference of binary dye solution was performed according to Adeyi et al. (2019) [16], Idan et al. (2017) [19], and Wang et al. (2012) [20]. The components of a binary system MG and MB were measured, respectively, at $\lambda_{1,max}$ (617 nm) and $\lambda_{2,max}$ (665 nm), to give the absorptivity’s $A_{1}$ and $A_{2}$

(1) $$C_{MG}=\frac{\left(K_{MB2\;}A_{1})-(K_{MB1}\;A_{2}\right)}{\left(K_{MG1}\;K_{MB2})-(K_{MG2}\;K_{MB1}\right)}$$

(2) $$C_{MB}=\frac{\left(K_{MG1}\;A_{2})-(K_{MG2}\;A_{1}\right)}{\left(K_{MG1}\;K_{MB2})-(K_{MG2}\;K_{MB1}\right)}$$

$K_{MG1},\;K_{MG2}$, $K_{MB1}$, and $K_{MB2}$ represent the calibration constants for dyes MG and MB at $\lambda_{1,max}$ and $\lambda_{2,max}$, respectively. $C_{MG}$ and $C_{MB}$ denote the concentrations of MG and MB, respectively.

### 2.4. Characterization of Thiourea Modified Poly(AN-co-AA) (T-PAA)

FT-IR spectra of T-PAA were taken (before and after adsorption) using Fourier transform infrared spectrometer (Perkin Elmer, 1750X (PerkinElmer Inc., Waltham, MA, USA) by using potassium bromide (KBr) pellets in resolution range of 4000 to 400 cm^−1^ at room temperature. This FT-IR was performed to determine the surface functional groups of the modified polymer and ascertain the functional groups involved in the sequestration of cationic dyes. Scanning electron microscope (SEM) micrographs were acquired using a Hitachi S-3400N instrument (Hitachi Scanning Electron Microscope (SEM) (Hitachi S-3400N High-Technologies Corporation, Minato, Tokyo, Japan). It was operated at 10 to 20 kV to examine the morphology of T-PAA before and after adsorption process. To ascertain the percentage of carbon (C), hydrogen (H), nitrogen (N), and sulphur (S) contents in the polymer sample prepared, CHNS elemental analysis was done. CHNS Elemental micro-analysis was performed using LECO CHNS-932 (Leco Corporation, St. Joseph, MI, USA) spectrometer. Zeta potentials (surface charges) of T-PAA were measured by Zetasizer Nano Series (Malvern Panalytical Limited, Malvern Worcestershire, UK).

### 2.5. Fixed-Bed Column Experiments

The efficiency of T-PAA for MG and MB adsorption from binary solution was evaluated using designed laboratory scale continuous fixed-bed column. Fixed-bed adsorption column consist of cylindrical glass tower (internal diameter: 2.5 cm; height: 30 cm) packed with T-PAA adsorbent and coupled to a peristaltic pump (MasterFlex Console Drive, model 77521-47, Cole Parmer Instrument Company, Essex, USA). Prior to loading of T-PAA adsorbent, glass wool was fixed at the bottom of cylinder and then compacted using fine acid washed sea sand. The glass wool serves as packing to prevent adsorbent loss and provide even distribution flow across the column. T-PAA particles was then added to the column and packed with acid washed sand. The packed-bed was washed first with deionized water to avoid subsequent bed blocking. The T-PAA was compacted via natural gravity to form a uniform bed and complete expulsion of air bubbles. A binary mixture of MG and MB solutions were fed into column top with downward flow using peristaltic pump. The effluent aliquots were periodically withdrawn, and the supernatant MG and MB was scanned and measured. The initial pH, inlet dye concentration, mass of T-PAA, and flow rate were altered, respectively, as designed in Table 2 to investigate effect of column parameters.

Evaluation of the column performance based on the shape of the breakthrough curve is according to previous literatures [21,22]. The breakthrough curves were obtained from the plot of *C_t_/C_o_* versus time (*t*). The breakthrough point or time ($t_{B}$) and bed saturation/exhaustion time ($t_{e}$) chosen for this research were time when outlet concentration (*C_t_*) reached 50% and 90% of inlet concentration (*C_o_*), respectively. The total mass of MG and MB adsorbed, $q_{total}$ (mg), were calculated from the area under the breakthrough curve using Equation (3):

(3) $$q_{total}=\frac{Q}{1000}\int_{t=0}^{t=total}C_{ad}dt$$

where the adsorbed dye concentration and volumetric flow rate are denoted by $C_{ad}$ (mg/L) and Q (mL/min), respectively.

The experimental uptake capacity, $q_{B}$ (mg/g), is estimated by Equation (4), where $t_{B}$ is the breakthrough time (min) at 50% and $m_{ads}$ represent weight of T-PAA in the column (g).

(4) $$q_{B}=\frac{QC_{o}t_{B}}{m_{ads}}$$

### 2.6. Dynamic Adsorption Models

The design of an adsorption column requires precise prediction of concentration-time profile from breakthrough curve of discharged effluent from the column. The breakthrough time and curve shape (or slope) are key parameters, determining operations and dynamic response of adsorption in plug flow system. The Thomas, Bohart-Adams, and Yoon–Nelson equations were used to analyze the experimental data.

#### 2.6.1. The Thomas Model

Thomas model assumed that the sorption process follows Langmuir isotherm and pseudo-second-order kinetics of adsorption–desorption without axial dispersion [23,24]. The Thomas model is one of the most widely used model for describing adsorption process in a packed-bed tower. This model is expressed as:

(5) $$\frac{C_{o}}{C_{t}}=1+\exp\left(\frac{K_{TH\;}q_{o}\;m_{ads}}{Q}-K_{TH}\;C_{o}\;t\right)$$

The linear form of the model is given as

(6) $$\ln\left(\frac{C_{o}}{C_{t}}-1\right)=\frac{K_{TH}\;q_{o}\;m_{ads}}{Q}-K_{TH}\;C_{o}\;t$$

where $C_{o}$ and $C_{t}$ are the inlet and outlet MG concentrations. $K_{TH}$ represents the Thomas kinetic coefficient (mL/mg min); Q and t are volumetric flow rate (mL/min) and sampling flow time (min), respectively; and $q_{o}$ and $m_{ads}$ denote adsorption capacity (mg/g) and mass of T-PAA in the column (g), respectively.

#### 2.6.2. The Bohart-Adams Model

The Bohart and Adams model was derived based on the surface reaction theory with assumption that equilibrium is not instantaneous. Therefore, adsorption rate is proportional to residual capacity of adsorbent and concentration of adsorbate. The model was used to described the relationship between $\frac{C_{t}}{C_{o}}$ and t in a plug flow system for the sorption of chlorine on activated charcoal [25]. It established a correlation between time and bed depth of the column and expressed as

(7) $$\frac{C_{o}}{C_{t}}=1+\exp\;\left(\frac{K_{AB}\;N_{o}\;Z}{U_{o}}-K_{AB}\;C_{o}\;t\right)$$

Linearized form of the Bohart–Adams equation can be written as

(8) $$\ln\;\left(\frac{C_{o}}{C_{t}}-1\right)=\frac{K_{AB}\;N_{o}\;Z}{U_{o}}-K_{AB}\;C_{o}\;t$$

$K_{AB}$, $N_{o}$, $U_{o}$, and Z represent Bohart–Adams kinetic coefficient (L mg^−1^ min^−1^), saturation concentration (mg L^−1^), superficial velocity (cm min^−1^), and bed depth (cm), respectively.

#### 2.6.3. The Yoon-Nelson Model

A simple model was also developed by Yoon and Nelson (1984) for analyzing the column’s breakthrough performance. The model is based on the assumption that the decreasing rate of the adsorption for each of the adsorbate particle is directly proportional to both the adsorbate adsorption and the adsorbate breakthrough on the adsorbents [26]. Yoon-Nelson model required no elaborate details or data concerning the characteristics of adsorbate, type of adsorbent or its physical features. The Yoon and Nelson model is given by

(9) $$\frac{C_{o}}{C_{t}}=1+\exp\;\left(\tau \;K_{YN}-K_{YN}\;t\right)$$

The Yoon-Nelson model is linearized for a single component system and expressed as

(10) $$\ln\;\left(\frac{C_{t}}{C_{o}-C_{t}}\right)=K_{YN}\;t-\tau \;K_{YN}$$

The Yoon-Nelson rate constant is denoted by $K_{YN}$ (min^−1^), τ is the required time for 50% adsorbate breakthrough (min), and t is the sampling time (min).

The dynamic adsorption model parameters were determined by fitting of the three models with experimental data through linear regression. The superiority or suitability of each model was measured via coefficient determination (*R*^2^) and analysis of error.

### 2.7. Column Regeneration

Experimental study on the possibility of desorbing MG and MB ions from T-PAA adsorbent is highly important for potential industrial application. Seven regeneration cycles on adsorption-regeneration were performed for adsorbed dye-loaded T-PAA polymer. The mixed solution of 1.0 M HNO_3_ and 0.5 M thiourea was used as eluent at 3 mL/min flow rate for 30 min. Post-regeneration, the T-PAA polymer was washed with distilled water for 10 min and reused in the next cycle of the column binary adsorption study.

## 3. Results and Discussion

### 3.1. Surface Characterization of the Synthesized T-PAA

**Figure 3:**
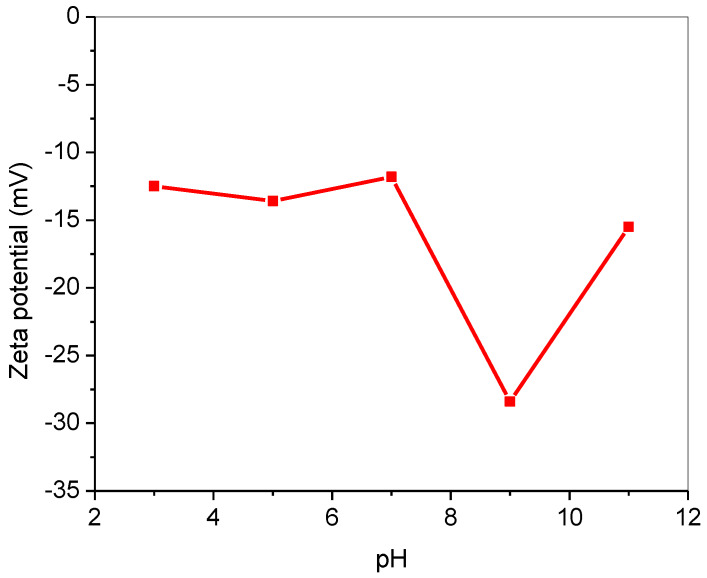
Zeta potential of thiourea-modified poly(acrylonitrile-*co*-acrylic acid) (T-PAA).

Detail morphological and physiochemical characterization of T-PAA adsorbent has been reported in our previous studies [13,14,15]. Table 3 shows the surface characterization and micro-elemental analysis of the T-PAA polymeric adsorbent. According to International Union of Pure and Applied Chemistry (IUPAC) classification, T-PAA is mesoporous with 47.93 nm average pore diameter [27,28,29]. T-PAA was negatively charged in acidic, alkaline, and neutral conditions, based on the zeta potential measurement, as indicated by Figure 3. The values of zeta potentials of T-PAA negatively increased with increases in the solution pH from 3 to 9. The variations in zeta potential at every pH strongly support the successful modification of the poly(acrylonitrile-co-acrylic acid) by thiourea [30,31,32]. This showed the existence of negatively charged functional groups at the edges of T-PAA, as thioamide, carbonyl, and hydroxyl groups leads to hydrophilic/hydrophobic balance with negative charge density [33,34]. Catherine and coworkers, in 2018, reported similar trends of higher negative surface charge of graphene oxide (GO) nanoflakes at higher pH [34]. This indicates that T-PAA was stable and confirmed the presence of negatively charged functional groups on the edges of T-PAA surface that enhanced the adsorptive activity.

**Figure 4:**
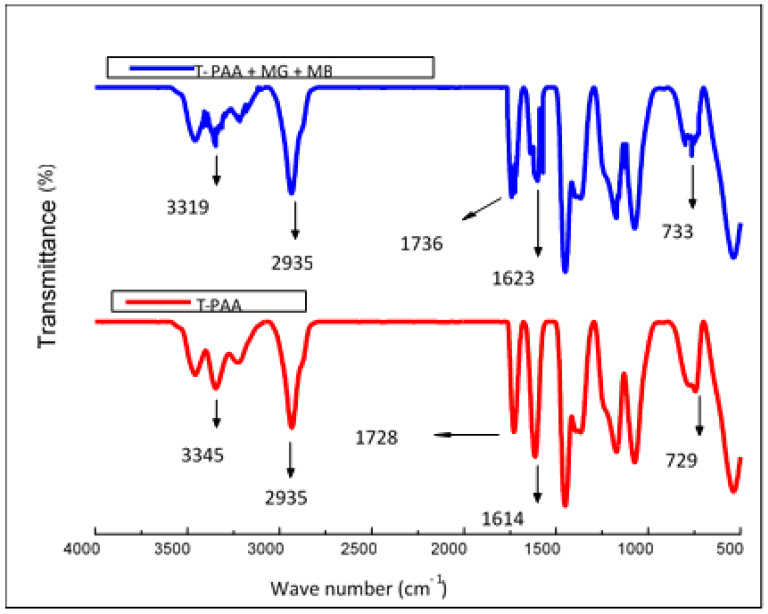
FTIR spectra of synthesized T-PAA (red), and T-PAA loaded with MG and MB dyes (blue).

The FTIR spectra (Figure 4) indicated that the T-PAA adsorbent was posed with prominent IR stretching peaks at around wave numbers 3345 cm^−1^, 2935 cm^−1^, and 1614 cm^−1^, which, respectively, were due to presence of –OH/–NH_2_, –CH_2_, and –C=N– vibrations [30,31,32]. Besides, –C=S bands was also prominent at 1015 cm^−1^ and 729 cm^−1^. This confirmed the successful introduction of thioamide groups into the adsorbent surface. Apparently, the shift in transmittance from 3345 cm^−1^, 729 cm^−1^ to 3319 cm^−1^, 733 cm^−1^ confirmed that the thioamide and hydroxyl group functionalities of T-PAA were involved in the entrapment of MG and MB dyes. This is due to existence of strong interactions between dye cations and anionic T-PAA (consisting probably of inner sphere surface complexation).

**Figure 5:**
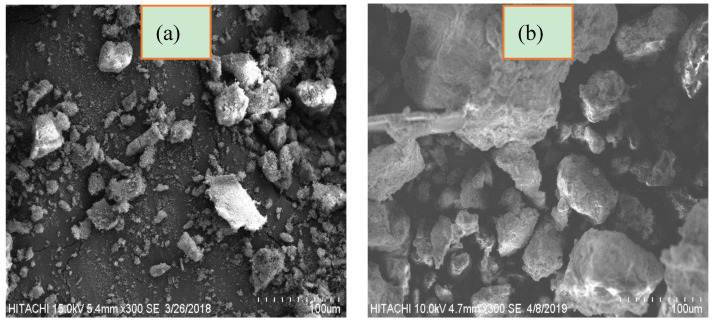
SEM micrographs of (**a**) T-PAA and (**b**) T-PAA loaded with MG and MB dyes.

Figure 5 presents the SEM images of T-PAA before and after cationic dyes sequestration. Prior to adsorption, T-PAA exhibits corrugated surface and irregular pores/shapes. After adsorption, a compactly packed and accumulated morphology could be observed with no any perceivable voids, signifying that MG and MB cations were entrapped via a pore-filling mechanism.

### 3.2. Effect of Initial pH

**Figure 6:**
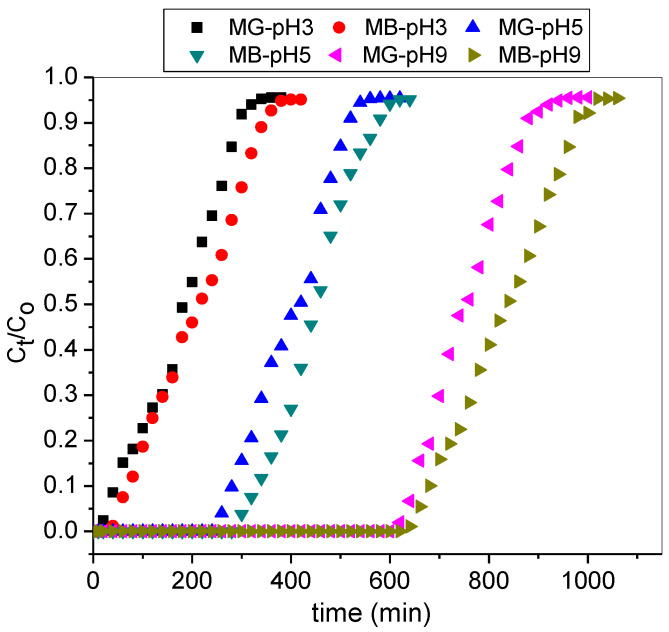
Breakthrough curves for adsorption of MG and MB in the binary system at varied solution pH ($C_{0}$: 50 mg/L; Z: 6 cm; Q: 3.0 mL/min).

The influence of solution pH on the percentage removal of cationic MG and MB in a binary system onto T-PAA were explored at varied pH (3, 5, and 9), while concentration, flow rate, and bed depth were kept constant. The breakthrough curves for MG and MB are, respectively, presented in Figure 6.

The T-PAA breakthrough bed capacity was minimum at pH 3 (MG: 5.4 mg/g; MB: 6.0 mg/g) and maximum at pH 9 (MG: 22.8 mg/g; MB: 24.9 mg/g) as reported in Table 4. This is due to the fact that MG and MB dye exists in the dissociated form in aqueous solutions as cationic dye ions. At low pH, H^+^ compete with dye ions, causing a reduction in the uptake of both dyes from the liquid phase. However, with an increase in solution pH, the negatively charged surface of T-PAA increased. This condition enhanced the dye uptake due to the electrostatic force of attraction between the negative sorption sites of adsorbent and cationic dye. These findings are supported by Alqadami and co-workers who have reported the removal of MG and MB by metal-organic framework (MOF) nanocomposite [35].

### 3.3. Effect of Inlet Dyes Concentration

**Figure 7:**
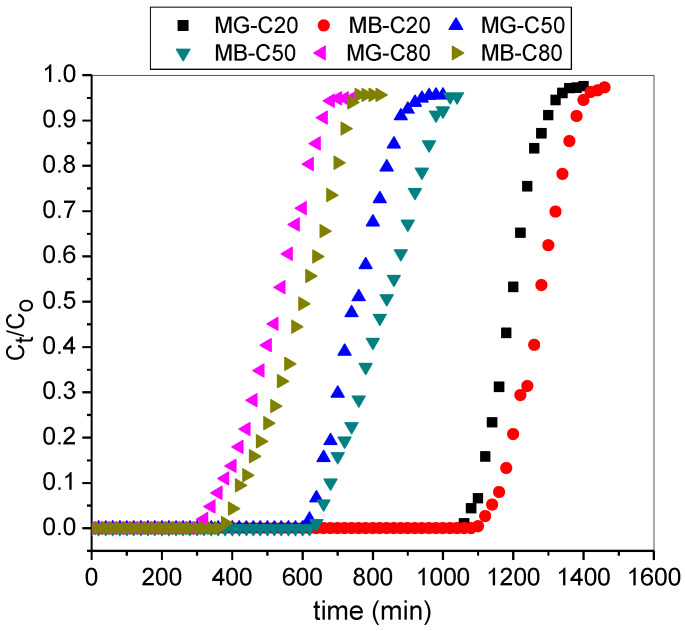
Breakthrough curves for adsorption of MG and MB in the binary system at varying initial concentrations (pH: 9; Z: 6 cm; Q: 3.0 mL/min).

The change in the influent dye concentration had a notable effect on the breakthrough curves as illustrated in Figure 7. It was observed that breakthrough and saturation time was reached earlier when the concentration of the binary dye mixture was higher. Additionally, at high inlet concentrations, the breakthrough curves appeared steeper compared to lower dye concentration with shorter mass transfer zone. The results demonstrated high bed capacity with an increase in initial dye concentration due to the change in the rate of adsorption. Therefore, the adsorption of binary cationic dyes at breakthrough and exhaustion were quite greater at high concentrations because higher concentrations effect a stronger driving force and smaller mass resistance for dye uptake in a continuous column study. This phenomenon was also reported by López-Cervantes et al. (2017) and Idan et al. (2017) for column adsorption of azo dye and anionic acid dye, respectively [36,37].

### 3.4. Effect of Adsorbent Bed Height

**Figure 8:**
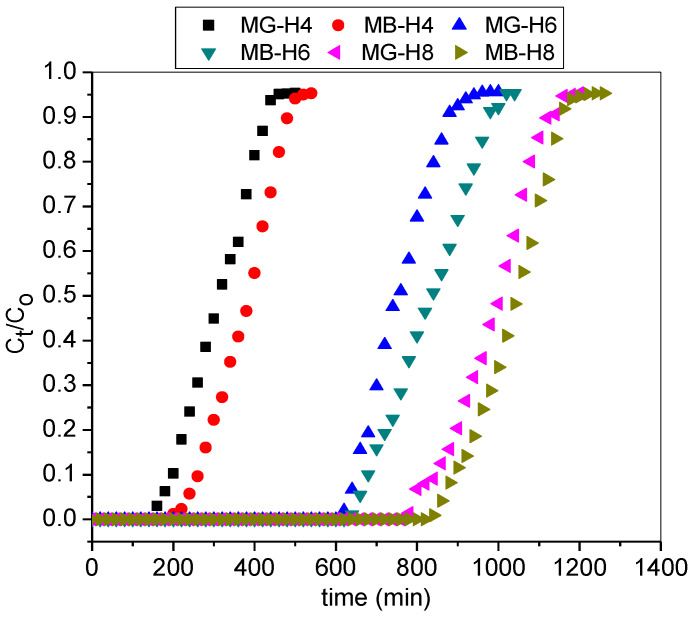
Breakthrough curves for adsorption of MG and MB in the binary system at varied bed height (pH: 9; $C_{0}$: 50 mg/L; Q: 3.0 mL/min).

The influence of column bed height (mass of T-PAA) on binary adsorption of cationic dye was investigated. Figure 8 shows the breakthrough curves as a function of time at varied bed height (4, 6, and 8 cm). As depicted by Table 4, the breakthrough and saturation time increased with increasing bed depth. The breakthrough time increases from 310 min to 1010 min, and 380 min to 1040 min, respectively, for MG and MB when bed depth increases from 4 cm to 8 cm. This is due to an increase in the total number of binding sites (for dye entrapment) with higher bed height which contains more T-PAA adsorbent [38]. Insufficient diffusion time is associated with a column with reduced or lower bed depth, resulting in lower values of bed capacities. The result obtained exhibit enhanced column performance at higher adsorbent load. The same result phenomenon has been reported also by Nath et al. (2016) for MG uptake using calcium alginate immobilized *Baccillus cereus* adsorbent [39].

### 3.5. Effect of Influent Flow Rate

**Figure 9:**
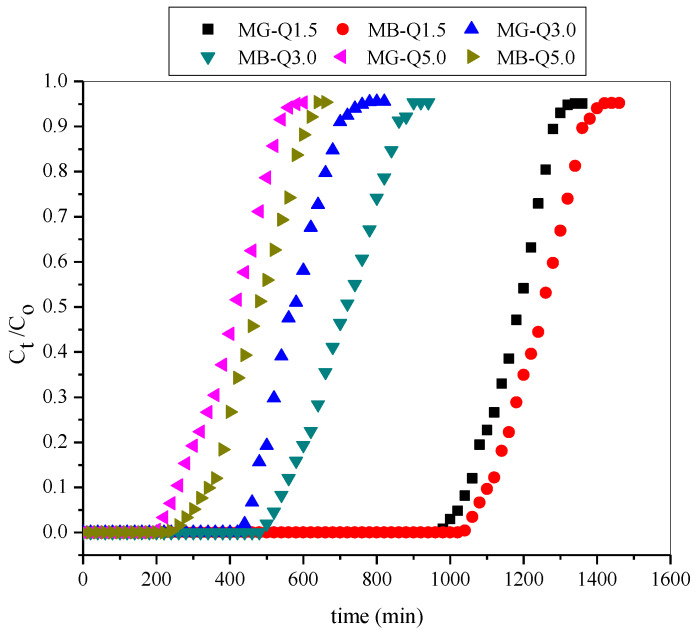
Breakthrough curves for adsorption of MG and MB in a binary system at a varied flow rate (pH: 9; $C_{0}$: 50 mg/L; Z: 6 cm).

Figure 9 shows the breakthrough curves with respect to time at three distinct flow rates (1.5, 3.0, and 5.0 mL/min). Table 4 depicts lower breakthrough and saturation time at a higher flow rate. At 5.0 mL/min, $t_{B}$ was 480 min for MG and 530 for MB, respectively, while this time was 1190 min and 1250 min when the flow rate was 1.5 mL/min. Similar result trends were also observed and reported by Charola et al. (2018) [40] and Zhou et al. (2015) [22]. Obviously, the increment in the residence time distribution of the bi-solute in the column results in longer contact time between adsorbate and T-PAA, therefore, equilibrium was attained. Conversely, the breakthrough bed capacities were high at 5.0 mL/min. This probably suggests that desorption took place at longer residence time.

Overall, the slight variation in the preference for individual cationic dye with respect to $q_{B}$ and $q_{sat}$ at all column parameters (as observed from Table 4), indicates that T-PAA has more affinity towards MB than MG. The triangular and linear molecular structures (Figure 1) of MG and MB, respectively may account for preferential dye uptake.

### 3.6. Application of Thomas Model

The values of Thomas model constant, $K_{TH}$, $q_{0}$, and statistical parameters ($R^{2}$, SSE) were calculated using linear regression analysis (Figure S1) and are presented in Table 5. The values of $K_{TH}$ slightly declined as the bed height increased from 4 to 6 cm. This decrement in $K_{TH}$ value is probably linked to the mobility of MG and MB ions within the mass transfer zone. Table 5 shows that the adsorption capacity $q_{0}$ increased with initial dye concentrations and T-PAA load. The calculated bed capacity $q_{0}$ was 25.32 mg/g (MG) and 28.51 mg/g (MB) at pH 9, 6 cm bed depth, 3.0 mL/min flow rate, and 80 mg/L initial binary dye concentration.

The experimental breakthrough bed capacity, $q_{B}$, was in good agreement with $q_{0}$ predicted by the Thomas model. Additionally, the value(s) of the correlation coefficient, $R^{2}$ at different column operating conditions, ranges between 0.95 and 0.99 for both cationic dyes and found to be significant statistically at the 95% confidence level. This signifies that binary cationic dye entrapment process is controlled by mass transfer at the interface, characterized by monolayer adsorption and yet not limited by chemical reaction [37,41].

### 3.7. Application of Bohart-Adams Model

Table 6 summarized the Bohart-Adams model constants and statistical factors that were deduced from the gradient and intercept of the linear model fittings (Figure S2) for binary MG and MB uptake by T-PAA in a column mode. It is observed that the value of $K_{BA}$ declined with an increase in bed height and dye inlet concentrations. This might be associated with the dominance of the external mass transfer at the initial part of the dye sorption process. Lower bed depth as well as lower concentration cause entrapment of more dye in the column. This outcome is supported by the research findings of [42,43,44]. The correlation coefficient value ($R^{2}$: range between 0.77 and 0.99) and SSE value are comparable to the corresponding values obtained from Thomas model. This suggests that the Bohart-Adams model is also suitable for the process description.

### 3.8. Application of Yoon-Nelson Model

Figure S3 shows the linear fitting of the experimental data into the Yoon and Nelson model (Equation (10)). The constants of the Yoon-Nelson model and statistical parameters at various operational conditions are summarized in Table 7.

Notably, the Yoon–Nelson model constant $K_{YN}$ decreased with increasing initial dye concentrations and bed depth. This is attributed to mass transfer resistance during cationic dye entrapment. While the predicted breakthrough time, τ increased as pH and T-PAA load increases, which is linked to the electrostatic attraction between the positively charge adsorbate and anionic polymeric adsorbent surface as well as the availability of more sorption site [45]. It was observed from Table 7, that the experimental breakthrough time $t_{B}$ and predicted breakthrough time agreed well, and the model applicability was supported by high values of correlation coefficient ($R^{2}\geq 0.95)$.

Overall, according to the values of linear regression coefficients $R^{2}$ and sum of square errors, SSE listed in Table 5, Table 6 and Table 7 for the entire breakthrough curves, the three models had a good account and suitable descriptions of the adsorption data at various column operating conditions. This confirms the applicability of the three models to column design and analysis. A similar behavior was report by [46]. All the three models were also reported valid for binary adsorption of anionic dyes, acid blue 25 (AB) and acid green 25 (AG) by [37].

### 3.9. Regeneration of T-PAA Adsorbent

**Figure 10:**
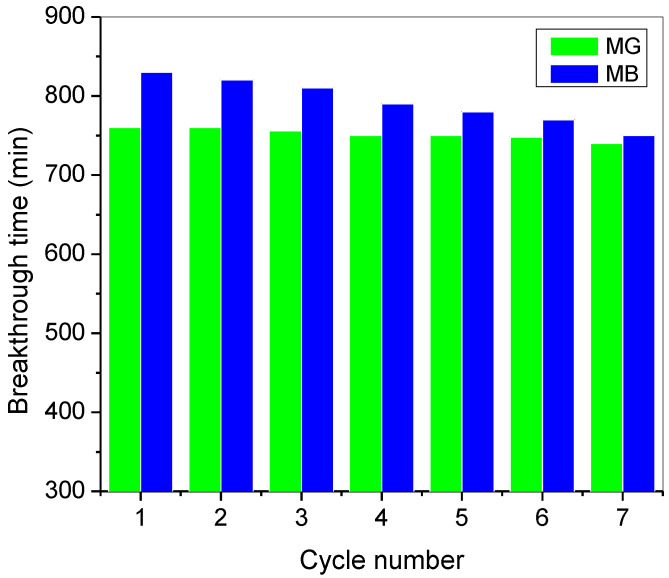
Regeneration performance of T-PAA at consecutive cycle in MG and MB binary dye system (pH: 9; $C_{0}$: 50 mg/L; Z: 6 cm; Q: 3.0 mL/min).

The regeneration and reusability of adsorbent materials is a vital factor in industrial scale viability. The column regeneration of dye-saturated T-PAA adsorbent was performed using a mixture of nitric acid and thiourea as best eluent according to [14,15]. Figure 10 shows the respective breakthrough (corresponding to 50% of influent concentration) time at successive regeneration cycle. At the end of the seventh cycle, the regenerated efficiencies of T-PAA were dropped to 81.1% and 75.3% for MG and MB, respectively, concerning the fresh adsorbent. The regenerated polymeric adsorbent demonstrates the capacity for multiple usages with little reduction in breakthrough time. This reduction in regeneration efficiency is associated with the difficulty in driving the elution process to completion; therefore, more binding sites were occupied by the accumulated non-desorbed dye ions [47,48]. This results show that T-PAA has an outstanding potential for entrapment of cationic dyes after seven adsorption–desorption cycles.

## 4. Conclusions

In this work, a thiourea-modified poly(acrylonitrile-co-acrylic acid) (T-PAA) adsorbent was applied to remove cationic MG and MB dye simultaneously in a fixed-bed column. The effect of solution pH, inlet dye concentration, flow rate, and sorbent bed depth on binary adsorption process was investigated, and the experimental breakthrough curves were obtained. It was observed that the dye removal capacity found to significantly affect the shape of breakthrough curves and the rate of adsorption by the various column operation parameters. The result demonstrated that moderate concentration of dyes, suitable flow rate, high pH, and bed depth are vital for higher sorption efficiency. In comparison, experimental breakthrough data were well fitted by Thomas, Bohart-Adams, and Yoon-Nelson dynamic models. Furthermore, reusability study conducted in continuous column operations revealed that T-PAA adsorbent can be repeatedly used for removal of dye from liquid phase after seven adsorption-elution cycles. Based on experimental findings, the T-PAA polymer proved to be valuable and regenerable adsorbent towards the separation of cationic dyes from the binary solution, and possible water reuse in the industry or irrigation purpose.

## Figures and Tables

**Table 1:** General properties of cationic MG and MB dyes.

Name of the Commercial Dye	Malachite Green, MG	Methylene Blue, MB
Color Index Name	Basic Green 4	Basic Blue 9
λ _max_ (nm)	617	665
Molecular Weight (g/mol)	364.92	319.85
Charge	(+)	(+)
Chemical Formula	C_23_H_25_ClN_2_	C_16_H_18_ClN_3_S

**Table 2:** Column experimental conditions during the binary adsorption of MG and MB dye onto T-PAA.

Influent pH	Initial Concentration (mg/L)	Bed Depth (cm)	Flow Rate (mL/min)
3, 5, 9	50	6	3
9	20, 50, 80	6	3
9	50	4, 6, 8	3
9	50	5	1.5, 3.0, 5.0

**Table 3:** Surface characterization of the T-PAA adsorbent.

Physiochemical Features	T-PAA
BET surface area (m^2^/g)	26.31
Total pore volume (cm^3^/g)	0.158
Average pore size (nm)	47.93 (mesoporous material)
Carbon (wt%)	61.94
Hydrogen (wt%)	5.618
Nitrogen (wt%)	25.06
Sulphur (wt%)	3.187

**Table 4:** Column adsorption data for MG and MB onto T-PAA in the binary system.

Dye	pH	C_o_ (mg/L)	Z (cm)	Q (mL/min)	t_B_ (min)	t_e_ (min)	$q_{B\;}(\mathrm{mg}/g)$	$q_{sat}\;(\mathrm{mg}/g)$
MG	3	50	6	3.0	180	340	5.40	10.20
	6	50	6	3.0	420	560	12.60	16.80
	9	50	6	3.0	760	950	22.80	28.50
	9	20	6	3.0	1200	1340	14.40	16.08
	9	80	6	3.0	530	720	25.44	34.56
	9	50	4	3.0	310	450	15.50	22.50
	9	50	8	3.0	1010	1170	21.64	25.07
	9	50	6	1.5	1190	1340	17.85	20.10
	9	50	6	5.0	480	660	24.00	33.00
MB	3	50	6	3.0	200	380	6.00	11.40
	6	50	6	3.0	450	620	13.50	18.60
	9	50	6	3.0	830	1020	24.90	30.60
	9	20	6	3.0	1270	1410	15.24	16.92
	9	80	6	3.0	600	760	28.80	36.48
	9	50	4	3.0	380	510	19.00	25.50
	9	50	8	3.0	1040	1200	22.29	25.71
	9	50	6	1.5	1250	1440	18.75	21.60
	9	50	6	5.0	530	720	26.50	36.00

**Table 5:** Thomas model constants and statistical parameters for MG and MB adsorption by T-PAA at different column conditions.

Column Variables					Thomas				
Dye	pH	C_o_ (mg/L)	Z (cm)	Q (mL/min)	$K_{TH}\times 10^{-4}\;(L/\min\;\mathrm{mg})$	$q_{o}\;(\mathrm{mg}/g)$	$q_{B}\;(\mathrm{mg}/g)$	$R^{2}$	SSE
MG	3	50	6	3.0	3.54	5.49	5.40	0.975	1.72
	5	50	6	3.0	3.31	12.23	12.60	0.962	2.68
	9	50	6	3.0	3.40	23.08	22.80	0.947	4.08
	9	20	6	3.0	0.12	14.46	14.40	0.968	3.17
	9	80	6	3.0	1.96	25.32	25.44	0.987	1.11
	9	50	4	3.0	3.74	15.84	15.50	0.982	1.18
	9	50	8	3.0	3.15	21.28	21.64	0.983	1.48
	9	50	6	1.5	3.81	17.70	17.85	0.979	1.92
	9	50	6	5.0	3.45	23.61	24.00	0.957	3.91
MB	3	50	6	3.0	3.28	6.51	6.00	0.950	3.56
	5	50	6	3.0	3.52	13.69	13.50	0.986	0.95
	9	50	6	3.0	3.09	25.17	24.90	0.960	3.27
	9	20	6	3.0	0.11	15.38	15.24	0.973	3.02
	9	80	6	3.0	1.94	28.51	28.80	0.966	3.21
	9	50	4	3.0	4.61	19.26	19.00	0.932	8.36
	9	50	8	3.0	3.37	22.34	22.29	0.968	3.16
	9	50	6	1.5	3.54	18.74	18.75	0.958	4.03
	9	50	6	5.0	3.55	26.99	26.50	0.970	3.25

**Table 6:** Bohart-Adams model constants and statistical parameters for MG and MB adsorption by T-PAA at different column conditions.

Column Variables					Bohart-Adams				
Dye	pH	C_o_ (mg/L)	Z (cm)	Q	$K_{BA}\times 10^{-4}\;(L/\mathrm{mg}.\min)$	$N_{o}\times 10^{3}\;(\mathrm{mg}/L)$	$q_{B}\;(\mathrm{mg}/g)$	$R^{2}$	SSE
MG	3	50	6	3.0	2.00	7.15	5.40	0.9882	1.19
	5	50	6	3.0	3.44	9.69	12.60	0.9717	1.86
	9	50	6	3.0	3.40	14.74	22.80	0.9468	4.08
	9	20	6	3.0	5.00	13.68	14.40	0.7718	7.89
	9	80	6	3.0	1.96	19.50	25.44	0.9868	1.11
	9	50	4	3.0	3.74	9.63	15.50	0.9818	1.18
	9	50	8	3.0	3.15	15.99	21.64	0.9822	1.48
	9	50	6	1.5	3.81	14.75	17.85	0.9794	1.92
	9	50	6	5.0	3.44	17.20	24.00	0.9569	3.91
MB	3	50	6	3.0	3.20	7.87	6.00	0.9801	1.76
	5	50	6	3.0	3.40	12.43	13.50	0.9962	0.36
	9	50	6	3.0	2.95	17.86	24.90	0.9810	1.42
	9	20	6	3.0	6.00	12.63	15.24	0.7988	9.72
	9	80	6	3.0	1.97	20.51	28.80	0.9652	3.01
	9	50	4	3.0	4.61	12.94	19.00	0.9318	8.36
	9	50	8	3.0	3.37	16.92	22.29	0.9679	3.16
	9	50	6	1.5	3.54	15.62	18.75	0.9578	4.03
	9	50	6	5.0	3.55	19.99	26.50	0.9704	3.25

**Table 7:** Yoon-Nelson model constants and statistical parameters for MG and MB adsorption by T-PAA at different column conditions.

Column Variables					Yoon-Nelson				
Dye	pH	C_o_ (mg/L)	Z (cm)	Q (mL/min)	$K_{YN}\times 10^{-2}\;(\min^{-1})$	τ	$t_{B}\;(\min)$	$R^{2}$	SSE
MG	3	50	6	3.0	1.76	182	180	0.975	1.72
	5	50	6	3.0	1.65	408	420	0.962	2.68
	9	50	6	3.0	1.70	769	760	0.947	4.08
	9	20	6	3.0	2.31	1205	1200	0.968	3.17
	9	80	6	3.0	1.57	527	530	0.987	1.11
	9	50	4	3.0	1.87	317	310	0.982	1.18
	9	50	8	3.0	1.57	993	1010	0.983	1.48
	9	50	6	1.5	1.90	1180	1190	0.979	1.92
	9	50	6	5.0	1.72	472	480	0.957	3.91
MB	3	50	6	3.0	1.64	217	200	0.950	3.56
	5	50	6	3.0	1.76	456	450	0.986	0.95
	9	50	6	3.0	1.55	839	830	0.956	3.26
	9	20	6	3.0	2.24	1279	1270	0.973	3.02
	9	80	6	3.0	1.55	594	600	0.967	3.21
	9	50	4	3.0	2.31	385	380	0.932	8.36
	9	50	8	3.0	1.69	1042	1040	0.968	3.16
	9	50	6	1.5	1.77	1249	1250	0.958	4.03
	9	50	6	5.0	1.78	540	530	0.970	3.25

## Supplementary Materials

The following are available online at https://www.mdpi.com/1420-3049/25/11/2650/s1, Figures: Linear Regression Analysis for breakthrough curve modeling by Thomas model (Figure S1), Bohart-Adams model (Figure S2) and Yoon-Nelson model (Figure S3) for adsorption of MG and MB onto T-PAA in a binary solutions at different column conditions; (a) pH, (b) Concentration (mg/L), (c) Bed height, and (d) Flow rate mL/min.
