# Supplementary material for: Simultaneous Adsorption of Malachite Green and Methylene Blue Dyes in a Fixed-Bed Column Using Poly(Acrylonitrile-Co-Acrylic Acid) Modified with Thiourea
Source: Molecules. 2020 Jun 7;25(11):2650. doi: 10.3390/molecules25112650 (PMC7321146; doi:10.3390/molecules25112650)
Supplement: Supplementary file 1 [file molecules-25-02650-s001.pdf]

molecules

MDPI

Article

Simultaneous Adsorption of Malachite Green and Methylene Blue Dyes in a Fixed-Bed Column Using Poly(Acrylonitrile-Co-Acrylic Acid) Modified with Thiourea

Abel Adekanmi Adeyi [1,2], Siti Nurul Ain Md Jamil [3,4,*], Luqman Chuah Abdullah [1,5], Thomas Shean Yaw Choong [1,5], Kia Li Lau [1] and Nor Halaliza Alias [1,6]

1. Department of Chemical and Environmental Engineering, Faculty of Engineering, Universiti Putra Malaysia, UPM Serdang 43400, Malaysia; abeladeyi@abuad.edu.ng (A.A.A.); chuah@upm.edu.my (L.C.A.); csthomas@upm.edu.my (T.S.Y.C.); laukiali@hotmail.com (K.L.L.); norhalaliza@gmail.com (N.H.A.)
2. Department of Chemical and Petroleum Engineering, College of Engineering, Afe Babalola University Ado-Ekiti, ABUAD, KM. 8.5, Afe Babalola Way, Ado-Ekiti PMB 5454, Nigeria
3. Department of Chemistry, Faculty of Science, Universiti Putra Malaysia, UPM Serdang 43400, Malaysia
4. Centre of Foundation Studies for Agricultural Science, Universiti Putra Malaysia, UPM Serdang 43400, Malaysia
5. Institute of Tropical Forestry and Forest Products (INTROP), Universiti Putra Malaysia, UPM Serdang 43400, Malaysia
6. Faculty of Chemical Engineering, Universiti Teknologi MARA, 40450 Shah Alam, Selangor, Malaysia

* Correspondence: ctnurulain@upm.edu.my; Tel.: +6012-356-6076

Received: 13 May 2020; Accepted: 5 June 2020; Published: 7 June 2020

Supplementary Data

Model plots of the Thomas, Bohart-Adams and Yoon-Nelson column dynamic models of simultaneous adsorption of cationic dyes by Thiourea-modified Poly(acrylonitrile-co-acrylic acid) (T-PAA)

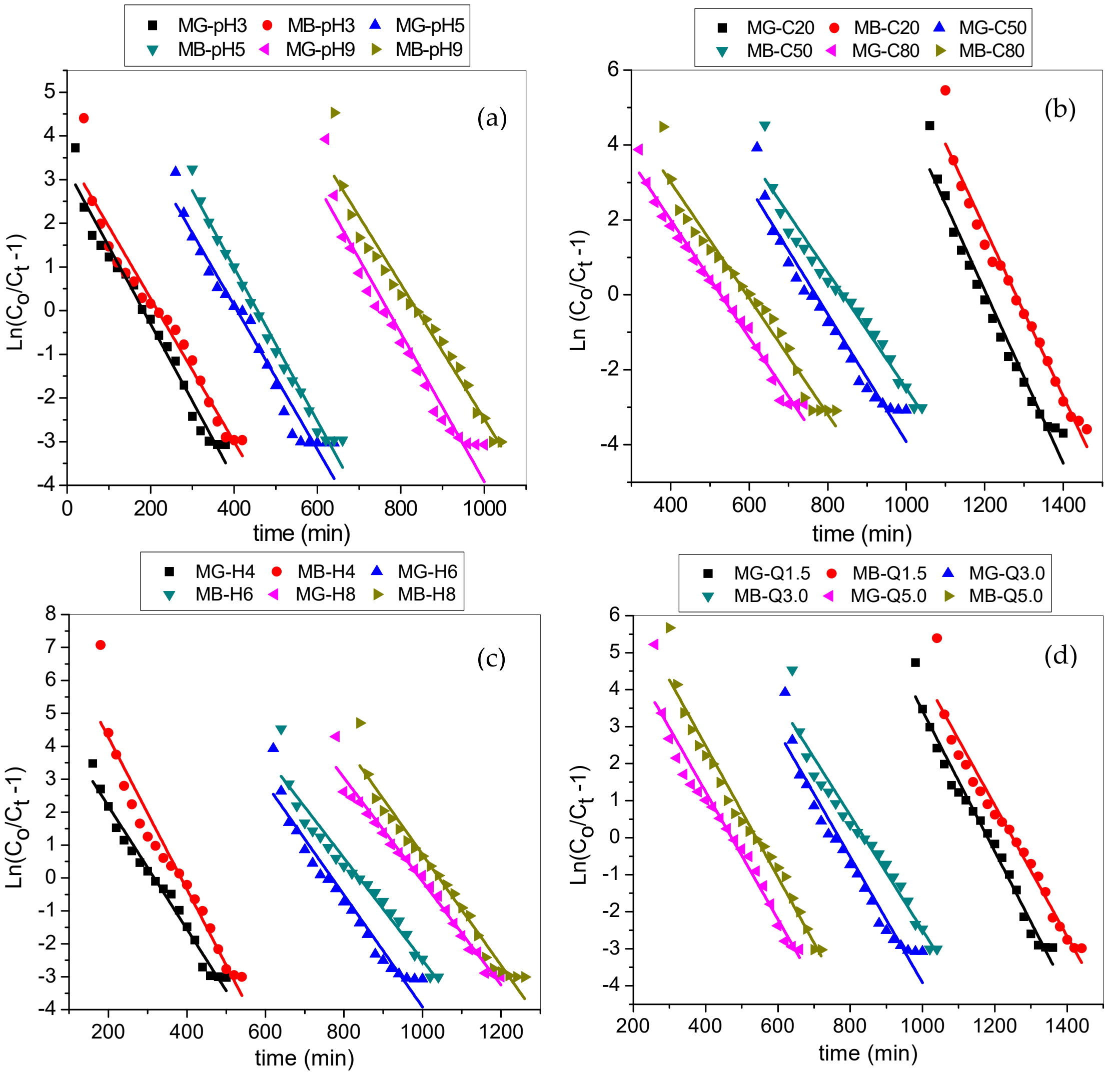

Figure S1: Linear Regression Analysis for breakthrough curve modeling by Thomas model for MG and MB onto T-PAA in a binary solutions at different column conditions; (a) pH, (b) Concentration (mg/L), (c) Bed height, and (d) Flow rate mL/min

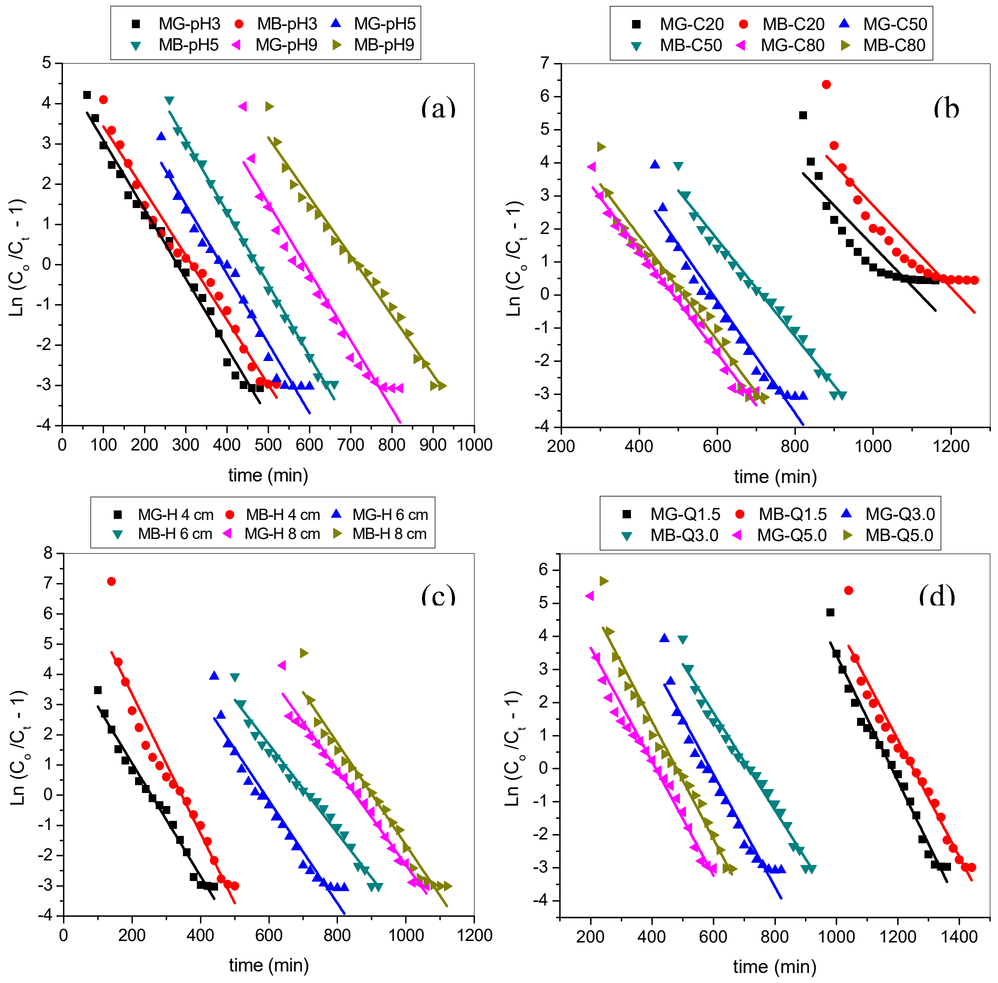

Figure S2. Linear Regression Analysis for breakthrough curve modeling by Bohart-Adams model for MG and MB onto T-PAA in a binary solutions at different column conditions; (a) pH, (b) Concentration (mg/L), (c) Bed height, and (d) Flow rate mL/min.

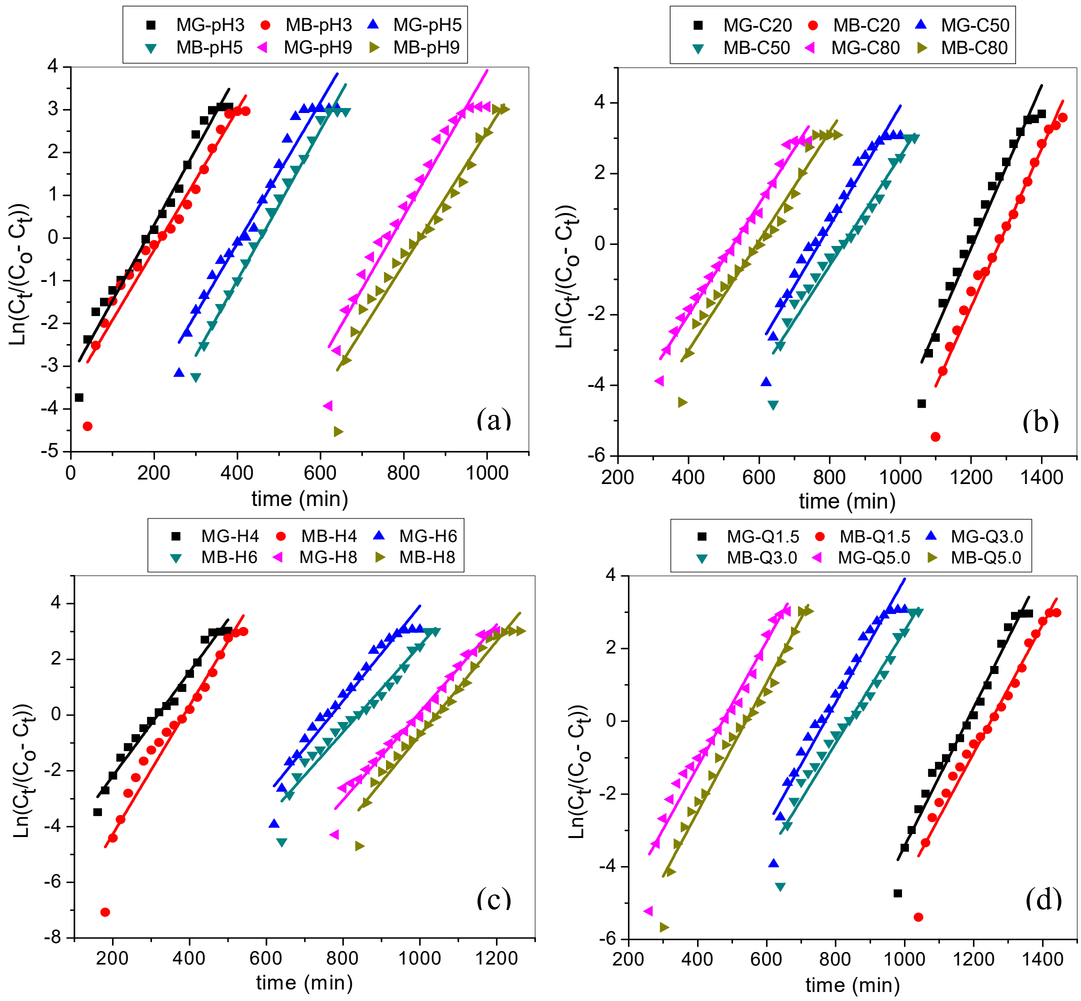

Figure S3. Linear Regression Analysis for breakthrough curve modeling by Yoon-Nelson model for MG and MB onto T-PAA in a binary solutions at different column conditions; (a) pH, (b) Concentration (mg/L), (c) Bed height, and (d) Flow rate mL/min

© 2020 by the authors. Licensee MDPI, Basel, Switzerland. This article is an open access article distributed under the terms and conditions of the Creative Commons Attribution (CC BY) license (http://creativecommons.org/licenses/by/4.0/).
